# Dose calculation for hypofractionated volumetric‐modulated arc therapy: approximating continuous arc delivery and tongue‐and‐groove modeling*

**DOI:** 10.1120/jacmp.v17i2.4989

**Published:** 2016-03-08

**Authors:** Jie Yang, Grace Tang, Pengpeng Zhang, Margie Hunt, Seng B. Lim, Thomas LoSasso, Gig Mageras

**Affiliations:** ^1^ Department of Medical Physics Memorial Sloan Kettering Cancer Center New York NY USA

**Keywords:** VMAT dose calculation, hypofractionated VMAT, gantry angle resolution, fluence resolution

## Abstract

Hypofractionated treatments generally increase the complexity of a treatment plan due to the more stringent constraints of normal tissues and target coverage. As a result, treatment plans contain more modulated MLC motions that may require extra efforts for accurate dose calculation. This study explores methods to minimize the differences between in‐house dose calculation and actual delivery of hypofractionated volumetric‐modulated arc therapy (VMAT), by focusing on arc approximation and tongue‐and‐groove (TG) modeling. For dose calculation, the continuous delivery arc is typically approximated by a series of static beams with an angular spacing of 2°. This causes significant error when there is large MLC movement from one beam to the next. While increasing the number of beams will minimize the dose error, calculation time will increase significantly. We propose a solution by inserting two additional apertures at each of the beam angle for dose calculation. These additional apertures were interpolated at two‐thirds’ degree before and after each beam. Effectively, there were a total of three MLC apertures at each beam angle, and the weighted average fluence from the three apertures was used for calculation. Because the number of beams was kept the same, calculation time was only increased by about 6%‐8%. For a lung plan, areas of high local dose differences (>4%) between film measurement and calculation with one aperture were significantly reduced in calculation with three apertures. Ion chamber measurement also showed similar results, where improvements were seen with calculations using additional apertures. Dose calculation accuracy was further improved for TG modeling by developing a sampling method for beam fluence matrix. Single element point sampling for fluence transmitted through MLC was used for our fluence matrix with 1 mm resolution. For Varian HDMLC, grid alignment can cause fluence sampling error. To correct this, transmission volume averaging was applied. For three paraspinal HDMLC cases, the average dose difference was greatly reduced in film and calculation comparisons with our new approach. The gamma (3%, 3 mm) pass rates have improved significantly from 74.1%, 90.0%, and 90.4% to 99.2%, 97.9%, and 97.3% for three cases, for calculation without volume averaging and calculation with volume averaging, respectively. Our results indicate that more accurate MLC leaf position and transmission sampling can improve accuracy and agreement between calculation and measurement, and are particularly important for hypofractionated VMAT that consists of large MLC movement.

PACS number(s): 87.55.kd

## I. INTRODUCTION

Volumetric‐modulated arc therapy (VMAT) delivers dose continuously, while gantry and multiple leaf collimator (MLC) leaves move dynamically with varying dose rate. The interplay of continuous gantry rotation, MLC motion, and dose‐rate modulation poses challenges with accurate dose calculation. The straightforward approach is to approximate the dynamic delivery with a set of closely spaced static beams with static gantry angles and MLC apertures.^1^, ^2^ The increase number of beams proportionally increases the dose calculation time for analytic dose calculation algorithms such as pencil beam convolution, collapsed cone convolution superposition, and linear Boltzman transport equation. However, it was previously shown that dose calculation accuracy does not depend on the number of beams used in arc approximation, but it depends on the MLC aperture and monitor units (MU) variation between the neighboring beams.^(3)^ While most commercial treatment planning systems impose a maximum limit on MLC displacement (i.e., limited aperture variation), there is no constraint for MLC motion for VMAT planning using our in‐house algorithm as an attempt to increase the degree of freedom in optimization. As a result, MLC displacement can be as large as 4 cm between adjacent beams or control points (CP) that are 2° apart. Up to 8% of dosimetric errors between calculation and film measurement have been observed in cases with large MLC motion or aperture variation. From the viewpoint of the target, Webb and McQuaid^(4)^ found that ray paths from a rotating gantry can be approximated as coming from fixed orientations with small angular spacing of 5°. Based on this finding, we have developed a method to improve dose calculation accuracy by simply modifying the fluence at each CP by taking into account the additional fluence from two interpolated MLC apertures between adjacent CPs.

Accurate MLC modeling, including tongue‐and‐groove effect and interleaf transmission, is another important aspect for hypofractionated VMAT dose calculation accuracy. Unlike IMRT delivery with a sliding window technique in which the leaves move only once across each treatment field and tongue‐and‐groove dose effects are small,^(5)^ during VMAT delivery a leaf may move back and forth repeatedly and extend into the radiation field for a prolonged time, thereby increasing such dose effects. Although studies have reported using collimator angles between 30° and 45° for VMAT,^(6)^ in some clinical situations 0° or 90° is desirable, based on our experience.^(7)^ For example, it is used in head and neck to facilitate field matching. In prostate and paraspinal tumor cases, a collimator angle of 90° can serve to better shield organs at risk such as rectal wall or spinal cord; in the latter case, maximum spinal cord dose can be reduced by 10% relative to a VMAT plan with the collimator angle at 0°. As we will show, however, VMAT using 90° collimator may become more sensitive to tongue‐and‐groove effects. Furthermore, for single or small number of fractions with high‐precision image‐guided patient setup as in hypofractionated VMAT cases, there is less smearing of the tongue‐and‐groove errors by patient setup uncertainties between fractions, compared to traditional IMRT plans with a large number of fractions.

**Figure 1:**
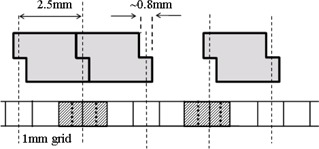
Illustration of volume averaging effects in sampling fluence through TG of HDMLC 2.5 mm wide leaves in 1 mm grid. The cross sections of three MLC leaves at the isocenter plane are shown. A section through 1 mm matrix is also shown. For fluence sampling purpose, parallel beams are illustrated in dotted lines. Volume averaging is required for the matrix elements filled with diagonal line pattern. Those elements are divided into two equal subelements by the dotted lines inside them for volume averaging.

Projecting to the isocenter level, the Varian Millennium MLC has 5 mm wide leaves within 10 cm of the beam central axis and 10 mm wide leaves at further distances. The width of tongue or groove is about 0.8 mm. A fluence resolution of 1 mm is used to align the fluence grid so that one 0.8 mm wide tongue or groove section contributes entirely to a single 1 mm wide matrix element in the direction perpendicular to the leaf travel direction. With this arrangement, the fluence seen by the center of the element can be assigned as the overall fluence for the entire element. For Varian Hi‐Definition MLC (HDMLC) that has 2.5 mm leaves, some tongue and groove sections are not modeled because of misalignments of the 1 mm grid with leaf boundaries, as illustrated in Fig. 1. The parallel beams are used to illustrate fluence sampling as fluence is only calculated at the isocenter level in our in‐house dose calculation algorithm. As a result, TG is not properly modeled and can cause dose errors. To correct this, we propose a volume‐averaging correction over the 1 mm wide element. Details are discussed in the next section.

In this study, we presented two methods to improve dose calculation accuracy for VMAT in the aspects of arc approximation sampling and TG modeling. These are particularly important because our in‐house optimization algorithm does not impose a maximum displacement constraint on the MLC. This results in VMAT plans with highly complex MLC motion, especially for hypofractionated cases, and modifications need to be made to the current dose calculation algorithm to account for these challenges.

## II. MATERIALS AND METHODS

### A. Dose calculation algorithm

The dose calculation algorithm of our in‐house treatment planning system is based on the pencil beam kernel convolution method.^(8)^ The effect of MLC is modeled by a two‐dimensional distribution of in‐air primary photon fluence, ∅(x,y), projected at the level of the isocenter. The fluence at point (x,y) is unity if the point is within the open portion of the field. The fluence becomes less than unity and is proportional to various MLC transmission factors depending on which part of the MLC leaf blocks the point at (x,y) (single tongue or groove of a leaf, mid‐part full thickness of leaf, or interleaf part). The dosimetric leaf gap is modeled with a transmission curve for rounded leaf end. Details of our MLC models are described by Chui et al.^(9)^

The dose for a given point (x,y) at depth d is calculated as

(1) $$D(x,y,d)=D_{0}(x,y,d)*\left[\frac{\iint \emptyset (x^{\prime },y^{\prime })K(x-x^{\prime },y-y^{\prime },d)dx^{\prime }dy^{\prime }}{\iint U(x^{\prime },y^{\prime })K(x-x^{\prime },y-y^{\prime },d)dx^{\prime }dy^{\prime }}\right]$$

where $D_{0}(x,y,d)$ is the dose for an open field with jaws only and it is calculated by using the empirical data tables of tissue maximum ratio, off‐axis corrections, and output factors.^(8)^ The ratio of the convolutions with the pencil beam kernel is used as correction factors for any additional beam modifying devices including the MLC. *U(x,y)* is a step function describing the uniform fluence distribution of the jaw‐defined field; *K(x,y,d)* is the pencil beam kernel at depth d in the medium; *d* is the equivalent depth that accounts for patient inhomogeneity and is calculated by ray tracing through the patient volume. The integral is done with Fast Fourier Transformation algorithm. The fluence is implemented as a 512×512 dimension matrix with a resolution of 1 mm. It should be noted that fluence is not a function of d. Therefore, calculation of fluence does not involve ray tracing in patient volume.

### B. Discretization of continuous arc delivery

For optimization, the continuous delivery arc of VMAT is discretized into a set of static beams or CP. Each CP is a snap shot of the gantry angle, MLC aperture, and accumulated MU during the dynamic motion. The optimization algorithm incorporates aperture‐based optimization with a progressive resolution scheme, similar to what was proposed by Otto.^(1)^ An optimization starts with a small number of CPs.^(2)^ As the optimization progresses through different stages, the number of CPs increases and the angular spacing between two adjacent CPs decreases. In the final stage, the angular spacing between two adjacent CPs is 2°. Our final dose calculation for plan evaluation uses a set of static beams with each beam corresponding to a VMAT CP in the final optimization stage. In our clinical implementation, there is no maximum displacement limit for the MLC. As a result, large MLC motion can occur in complex treatment plans. This leads to significant dose discrepancies when dose calculation was computed based on the original number of static beams (i.e., no interpolated beams involved). The simple solution is to increase the arc approximation resolution for dose calculation. However, as the number of beams increases, the computational burden proportionally increases and lengthens the calculation time. We solve this problem by accounting for the fluence from two additional apertures at each beam (i), where the additional apertures are interpolated from beam (i−1) and beam (i+1). With an angular spacing of the beams of 2°, the additional apertures are generated by interpolating the neighboring beams with an angular resolution of two‐thirds’ degree, which results in two interpolated apertures between the adjacent beams. Except for the first and last beam which has zero weight for dose calculation, the new fluence for the ith beam (i) is calculated as the average fluence of the three apertures:

(2) $$\emptyset_{i}^{New}(x,y)=(\emptyset_{i}(x,y)+\emptyset_{i}^{\left(1\right)}(x,y)+\emptyset_{i}^{\left(2\right)}(x,y))/3$$

Suppose $x_{\left(j,i\right)}$ is the leaf position of the jth leaf for the ith static beam, $\emptyset_{i}^{\left(1\right)}(x,y)$ is the fluence from the first new MLC aperture with each leaf position calculated by

(3) $$x_{j,i}^{\left(1\right)}=\frac{2}{3}*x_{j,i}+\frac{1}{3}*x_{j,i-1}$$

And $\emptyset_{i}^{\left(2\right)}(x,y)$ is the fluence from the second new aperture with each leaf position calculated by

(4) $$x_{j,i}^{\left(2\right)}=\frac{2}{3}*x_{j,i}+\frac{1}{3}*x_{j,i-1}$$

Suppose ${\text{MU}}_{i}$ is the accumulated MU at the ith beam, or CP. To calculate the relative MU (or relative weight) for each beam, we assign half of the delivered monitor units between the previous and the current beam and half between the current and the next beam as

(5) $$w_{i}=\frac{1}{2}*(MU_{i}-MU_{i-1})+\frac{1}{2}*(MU_{i+1}-MU_{i})$$

Effectively, each beam now contains information of three apertures at the same beam angle (one original aperture and two interpolated apertures at two‐thirds’ degree before and after the individual beam). Because the number of beams remains the same, the computational time is only minimally increased when the additional interpolated apertures are considered in dose calculation.

**Figure 2:**
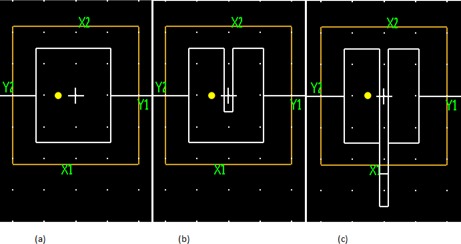
MLC apertures for the middle three control points of the simple arc are shown in white lines in (a), (b), and (c), respectively. Jaws are in yellow lines. White cross is the isocenter. Yellow circle is a point at 1 cm to the left of the isocenter. White dots are grid points on 2 cm×2 cm grid. The collimator angle of the arc is 90°.

For validation, we created a simple plan with short arc for a TrueBeam (Varian Medical Systems, Palo Alto, CA) equipped with HDMLC using 6 MV. There are a total of five CPs in the plan at gantry angle of 357°, 358°, 0°, 2°, and 3°, respectively. The accumulated dose is 0, 0.1, 0.5, 0.9, and 1 for the five CPs, respectively. The apertures for the second to fourth CPs are created, as shown in Fig. 2. Only the middle two leaf pairs (leaf pair number 30 and 31 for Varian HDMLC) are moving during delivery, with a total leaf travel of 4 cm for the trailing leaves and 8 cm for the leading leaves, respectively. Note that the first CP is the kick‐off beam, which has the same aperture shape as the second CP; the last CP is the ending beam, which has the same aperture shape as the fourth CP.

An ion chamber (cc01, IBA Dosimetry, Nuremberg, Germany) is used to measure the dose at the isocenter (marked as a cross in Fig. 2) and at 1 cm left of the isocenter (marked as a yellow circle in Fig. 2). Both points are measured at a depth of 5 cm in a Solid Water phantom. Measurements are compared to the calculation with one MLC aperture per beam, and the calculation with three MLC apertures per beam. The same measurements are used to validate our MLC modeling with volume averaging, which will be discussed in next section.

For additional validation, both the ion chamber and the film measurements are done for a selection of patient VMAT plans. Measurements are compared to the original calculation with one MLC aperture per beam, and the calculation with three MLC apertures per beam. The two different sets of calculations are referred to as Calc(1aper) and Calc(3apers), respectively. In addition, a third set of calculation is performed with original beams plus interpolated beams, with an interpolation resolution of two‐thirds’ degree (i.e., total number of beams is increased). Apertures and MU for the interpolated beams are linearly interpolated from the neighboring two original beams. This calculation is referred to as Calc(3×Beams).

Film measurements are done with rotational gantry using Gafchromic EBT3 films (ISP, Wayne, NJ), where the film is sandwiched between two slabs of 5 cm thick of Solid Water phantom placed on the couch. For chamber measurements, an IBA‐CC04 ion chamber is placed in a 2 cm thick of customized solid water slab that houses the chamber. A 5 cm thick slab is added to the top for buildup and another 5 cm thick slab is placed underneath for backscatter. Same as the film measurements, the phantom setup is placed on the couch and measurements are made with VMAT plans with rotating gantry. For ease of measurement, the ion chamber is set up to the isocenter. To minimize volume‐averaging effect and any error from chamber setup, the isocenter of all plans are adjusted such that they are located at a high‐dose region with minimal dose gradient. All VMAT plans for these measurements are planned and delivered on a Varian TrueBeam equipped with the Millennium MLC.

### C. Improving tongue‐and‐groove modeling with volume averaging

As described in the Materials & Methods section A, an MLC model with round leaf end and tongue‐and‐groove transmission was implemented with a 512×512 dimension fluence matrix with 1 mm resolution. Varian HDMLC has 2.5 mm wide leaves within 4 cm of the central axis and 5 mm wide leaves at further distance, while the width of tongue and groove is about 0.8 mm. On the 1 mm fluence grid that places the central axis at one element center, the tongue or groove sections of 2.5 mm wide leaves at 5 mm, 10 mm, 15 mm, etc., from the central axis are still contained in one single element. However, the tongue or groove sections at 2.5 mm, 7.5 mm, 12.5 mm, and so on from the central axis extend over two elements, as illustrated in Fig. 1. Thus, volume averaging over the 1 mm wide element is required. This is done by dividing each 1 mm element into two 0.5 mm wide subelements in the leaf width direction, and the fluences are computed at the center of each subelement. The average of the two fluences is then assigned to the given 1 mm element.

For validation the same simple arc with five CPs is used. The measurement is compared with calculation with and without volume averaging. Additionally, film measurements are done for three hypofractionated paraspinal VMAT plans delivered on a Varian TrueBeam linac with HDMLC. Measurements are compared to calculations with and without volume averaging over fluence matrix element.

## III. RESULTS

### A. Effect from using one aperture vs. using multiple apertures

For the simple five‐CP arc, the measurements and calculation results are summarized in Table 1. Calculation 1 is done with volume average over fluence matrix elements and the average fluence from three MLC apertures at each beam. Calculation 2 is done with volume average and the single MLC aperture fluence at each beam. For the isocenter which is under the moving leaves, the agreement between calculation and measurement is improved from 18% to 1%. In a real clinical case, this large discrepancy between calculation and delivery is unlikely to occur as the change in dose rate and aperture shape is reduced. For the point that is 1 cm left to the isocenter, there is no significant difference between two calculations. This is expected since there is no MLC leaf movement at this point.

**Table 1:** Chamber measurement compared to four different sets of calculations for a simple 5‐CP arc

	*Point at the Isocenter*		*Point at 1 cm Left of the Isocenter*	
	*Dose (cGy)*	*Ratio to Measurement*	*Dose (cGy)*	*Ratio to measurement*
Measurement	49.9	‐	86.0	‐
Calc 1^a^ (3ap+vol)	50.4	0.990	85.6	1.004
Calc 2^b^ (vol only)	42.3	1.180	85.4	1.006
Calc 3^c^ (3ap only)	51.3	0.971	85.8	1.001
Calc 4^d^ (no corr)	43.5	1.148	85.6	1.003

a ^a^ Calculation 1 is a calculation with both corrections of averaging fluence from three apertures and volume averaging of fluence over finer fluence resolution.

b ^b^ Calculation 2 is a calculation with only volume averaging correction.

c ^c^ Calculation 3 is a calculation with only three aperture average fluence correction.

d ^d^ Calculation 4 is with no correction.

**Figure 3:**
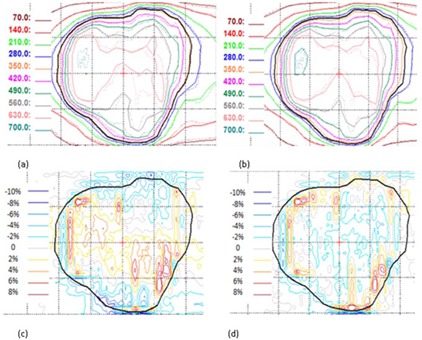
Dosimetric effects of one vs. three apertures at each beam. Panels (a) and (b) show the planar dose distributions in cGy from single aperture and three apertures overlay with film, respectively. The film measurement is in dotted lines and calculation is represented by solid lines. Panels (c) and (d) show the corresponding dose difference (film ‐ calculation) for (a) and (b), respectively. The dotted display grid size is 2 cm×2 cm.

For a hypofractionated lung VMAT arc, Figs. 3(a) and 3(b) show the planar dose overlay of film measurement to Calc(1aper) and Calc(3apers), respectively. Calculation is shown in solid lines and film measurement is shown in dotted lines. Figures 3(c) and 3(d) display the dose differences of film measurement and calculation (film ‐ calculation) for a given region of interest (ROI), which is represented by the accented black line. While the average dose difference is only 0.7%, discrepancies as large as 8% relative to the average dose of the ROI are seen in the comparison of film and Calc(1aper) in Fig. 3(c). Similarly, the average dose difference for the same ROI is −0.3% when comparing film to Calc(3apers), but area of large local dose difference (>4%) is reduced as shown in Fig. 3(d). For local gamma analysis (3%/3 mm), the passing rate for the film and Calc(1aper) comparison is 96.1%, while the passing rate is increased to 99.5% for the film and Calc(3apers) comparison.

The results for chamber measurements for 6 VMAT arcs are summarized in Table 2. In general, dose calculation accuracy is improved for Calc(3apers) and Calc(3×Beams). Calc(1aperes) shows the worst agreement with measurement. With only one aperture per CP for dose calculation, an absolute average dose error of 2.6% is observed in chamber measurements, with a maximum difference of 3.9%. Calc(3apers) shows better agreement to measurement with an absolute average difference of 1.3%, and a maximum dose difference of 2.7%. Calc(3×Beams) exhibits the same behavior, with an absolute average difference of 1.3% and a maximum difference of 2.5% compared to measurement. It is important to note that the dose distributions calculated in Calc(3apers) and Calc(3×Beams) are almost identical. This provides validation on small arc approximation and justifies our method of utilizing an average fluence from interpolated apertures at the same beam angle for dose calculation instead of adding interpolated beams. The calculation time for the additional apertures in Calc(3apers) is only increased by 6%‐8% compared to Calc(1aper), while the calculation time is increased by a factor of 3 for Calc(3×Beams), as shown in Table 3. All 6 VMAT arcs were delivered with regular Millennium MLC and do not require any volume averaging in tongue‐and‐groove modeling.

**Table 2:** Chamber measurement compared to three different sets of calculations for 6 VMAT arcs

		*Lung*			*Paraspinal*	
Dose (cGy)	Arc 1	Arc 2	Arc 3	Arc 4	Arc 1	Arc 2
Measurement	106.8	120.6	66.2	69.8	125.2	134.7
Calc (1aper)^a^	102.9	117.1	68.5	68.6	125.1	139.3
Calc(3apers)^b^	107.3	119.0	67.3	68.9	125.6	138.4
Calc(3×Beams) ^c^	107.2	118.9	67.4	68.9	125.6	138.2
*Ratio of Measurement to Calculation*						
Calc (1aper)^a^	1.039	1.030	0.966	1.018	1.001	0.967
Calc(3apers)^b^	0.995	1.014	0.983	1.013	0.996	0.973
Calc(3×Beams) ^c^	0.997	1.014	0.982	1.013	0.996	0.975

a ^a^ Calc(1aper) is calculation with original aperture at each beam.

b ^b^ Calc(3apers) is calculation with averaging fluences from the original aperture and two inserted interpolated apertures at each beam.

c ^c^ Calc(3×Beams) is calculation with tripled number of beams. Two additional beams are inserted linearly between two original beams.

**Table 3:** MLC leaf movement statistics of the VMAT arcs and calculation time

		*Lung*			*Paraspinal*	
Leaf movement of leaf pair 30 and 31	Arc 1	Arc 2	Arc 3	Arc 4	Arc 1	Arc 2
Average (cm)	0.8	0.5	0.5	0.6	0.2	0.7
SD (cm)	1.2	1.1	0.7	0.7	0.5	0.8
% of movement>2 cm	17	12	0	1	0	2
*Calculation Time Relative to Calc (1aper)* ^a^						
Calc(3apers)^b^	1.06	1.08	1.08	1.05	1.08	1.08
Calc(3×Beams) ^c^	2.9	3.0	3.1	3.0	3.0	2.8

a ^a^ Calc(1aper) is calculation with original aperture at each CP.

b ^b^ Calc(3apers) is calculation with averaging fluences from the original aperture and two inserted interpolated apertures at each CP.

c ^c^ Calc(3×Beams) is calculation with tripled number of beams. Two additional beams are inserted linearly between two original beams. Percent of movement greater than 2 cm is calculated by the number of leaf movements that are greater than 2 cm divided by total number of leaf movements.

To further investigate the differences occurring between the different sets of calculation and measurements, the average and the standard deviation (SD) of leaf movement are calculated (see Table 3). The MLC movement statistics are calculated for leaf pairs 30 and 31, which are located at 5 mm above and below the isocenter. These particular leaf pairs directly affect the isocenter dose and hence the chamber measurement and calculation comparisons. The leaf movement is defined as the absolute displacement of leaf positions between two successive beams. As shown in Table 3, there are large leaf movements in arcs 1 and 2 of the lung plans, which is reflected in the large dose discrepancies seen in Calc(1aper) and the improvement in Calc(3apers) and Calc(3×Beams). On the other hand, MLC motion for arc 2 in the paraspinal plan is moderate, and is deemed to be unlikely the main cause of the large dose differences between calculation and chamber measurement. The dose errors might be provoked by the location of isocenter, which is close to a high‐dose gradient.

### B. Effect of volume averaging in tongue‐and‐groove modeling for HDMLC

From Table 1, Calculation 1 is the calculation based on three apertures with volume averaging. Calculation 2 is only done with volume averaging and single MLC aperture fluence at each beam, while Calculation 3 is done with no volume average over the fluence elements, but with the average fluence from three MLC apertures at each beam for the simple 5‐CP case.

Calculation 4 is done with no volume average and with the single MLC aperture fluence at each beam. For the point that is 1 cm left to the isocenter, there is no significant difference between all four sets of calculations. This is expected since there is no leaf movement under this point throughout the entire delivery. At the isocenter, where the leaves are constantly moving, measurement is 1.0% and 2.9% lower compared to Calculation 1 and Calculation 3, respectively. Compared to Calculation 2 and Calculation 4, measurement is 18.0% and 14.8% higher, respectively. Calculation 1 shows a significant improvement in terms of agreement to measurement compared to Calculation 4, demonstrating that the additional aperture fluence information and the volume‐averaging correction are important to achieve accurate dose calculation for VMAT. Comparison of Calculations 1 and 3 shows that volume averaging yields a small, but nonnegligible, correction to tongue‐and‐groove effects in this simple 5‐CP case.

**Figure 4:**
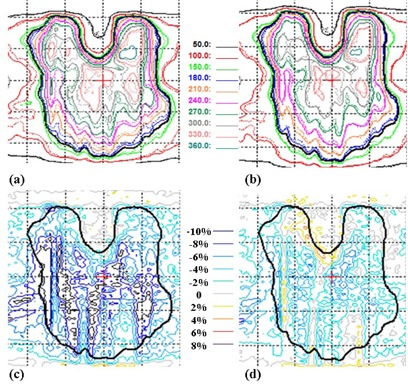
Comparison of dose calculations with film measurement for paraspinal case 1 with HDMLC. Panels (a) and (b) show the planar dose distribution in cGy calculated without volume‐averaging correction and with volume‐averaging correction, respectively. The film measurement is in dotted lines and calculation is represented by solid lines. Panels (c) and (d) show the corresponding dose difference as a percentage of the average dose (film ‐ calculation) for (a) and (b), respectively. The thick black line defines the ROI for average dose and gamma pass rate. The dotted display grid size is 2 cm×2 cm.

For hypofractionated paraspinal VMAT case 1, Fig. 4(a) shows the isodose overlay of film measurement to dose calculation without volume averaging and the dose difference (film ‐ calculation) is shown in Fig. 4(c). Similarly, Figs. 4(b) and 4(d) show the comparison between film measurement and calculation with transmission averaging. Both sets of calculation (with and without transmission volume averaging) are computed with 3 apertures per beam. Without volume averaging in dose calculation, the local gamma (3%, 3 mm) pass rate is 74.1%, with a difference of −6.1% in average dose calculated in the ROI when compared to film. The large dose errors occur because some tongue‐and‐groove sections are completely missed during MLC modeling in calculation. With volume averaging, the difference in the average dose between film and calculation is significantly reduced to −1.9%, with a gamma passing rate of 99.2%. Similar results are observed in two other cases. The difference in average dose in the ROI is improved from −6.2% and −6.3% to −1.5% and −2.8% for film compared to calculation without volume averaging and calculation with volume averaging, respectively. Gamma passing rate was also improved from 90.0% and 90.4% to 97.9 and 97.3%, respectively.

## IV. DISCUSSION

In this study, we present a simple scheme to improve dose calculation accuracy for hypofractionated VMAT plans with large MLC motion. By accounting for the fluence from the additional interpolated apertures at the same given planned CP or beam angle, the same dose calculation accuracy is achieved as the calculation computed with interpolated beams. Because the number of beam angles remains the same, the CPU time spent on calculating fluences from multiple apertures is only a fraction of the time needed to calculate ray tracing through the patient volume at multiple gantry angles. For the cases used in this study, about two‐thirds of CPU time is saved for Calc(3apers) compared to Calc(3×Beams). For cases with longer beam‐on time (i.e., higher MU) in combination with large MLC motion, more apertures can be interpolated to generate the average fluence for each beam. To correct for errors from undersampling MLC motion, Bedford^(10)^ proposed an approach to keep beam spacing at 5° and calculate with approximately 100 interpolated apertures between beams. Our approach is simpler by using only two interpolated apertures between beams while maintaining our beam resolution at 2°. Another difference in our approach is that, for each static beam angle, MLC apertures are interpolated from the current beam, and its preceding and following nearest neighbors, such that the gantry angle used for ray‐path calculation is centered in this small arc, whereas Bedford's method uses only two beams for interpolation and the gantry angle used for ray‐path calculation was the ending angle of the small arc.

Our findings show that, to more accurately account for tongue‐and‐groove transmissions, the fluence resolution should be comparable to the width of the actual tongue and groove, which is 0.8 mm for Varian MLC. Van Esch et al.^(11)^ also observed that smaller fluence resolution improved the dose calculation accuracy. In this study, 0.5 mm subelements are used to sample transmitted fluence through the MLC, and the average of each pair of subelements is assigned to the associated 1 mm element in the fluence grid, in order to improve dose calculation accuracy in the tongue and groove regions of the HDMLC. As mentioned in the Introduction, a collimator angle of 90° in paraspinal tumor cases can serve to better shield the spinal cord. In such plans, the leave edges are aligned with the spinal cord boundary in order to shield it at every gantry angle; thus, dose error accumulation from tongue‐and‐groove effects can occur. Our study of one example (Fig. 4) showed that average dose differences exceeding 6% can occur between film measurement and calculation without correct tongue‐and‐groove modeling. Since the dose calculation error occurs within the spinal cord region, it may affect maximum cord dose and misguide the dosimetrist because maximum cord dose is an important criterion for plan acceptance. We note that higher resolution may be used to model more detailed MLC features, such as 0.3 mm resolution used in version 10 of the AAA dose algorithm in Varian Eclipse system,^(11)^ albeit at the cost of increased CPU processing.

Although the two corrections described above are designed for our in‐house treatment planning system, they are not limited to our algorithm and can be applied to any calculation algorithms. It can also be applied in the dose calculation process during plan optimization.^12^, ^13^ This dose correction strategy does not sacrifice the optimization efficiency and is similar to the intermediate dose calculation approach in Varian Eclipse planning system that corrects for partial X‐ray scatter dose calculation used in optimization.^(13)^

## V. CONCLUSIONS

Two simple and efficient methods are described to improve dose calculation accuracy for hypofractionated VMAT plans. MLC aperture sampling at smaller than 1° of angular intervals can avoid major dose errors that occur from undersampling the largely varying MLC positions. Dose calculation accuracy is further improved by enhancing the TG modeling. For MLCs that have 5 mm and 10 mm leaf width, fluence resolution on the order of the TG width is desirable. Finer resolution may be required if the MLC leaf width is not a whole number in mm, such as 2.5 mm in HDMLC. Thus, optimal fluence resolution may depend on the MLC TG width and leaf width.

## ACKNOWLEDGMENTS

This research was funded in part through the NIH/NCI Cancer Center Support Grant P30 CA008748.

## COPYRIGHT

This work is licensed under a Creative Commons Attribution 4.0 International License.
